# Role of Complement in Multiorgan Failure

**DOI:** 10.1155/2012/962927

**Published:** 2012-12-20

**Authors:** Daniel Rittirsch, Heinz Redl, Markus Huber-Lang

**Affiliations:** ^1^Division of Trauma Surgery, Department of Surgery, University Hospital Zurich, Raemistrasse 100, 8091 Zurich, Switzerland; ^2^Ludwig Boltzmann Institute for Experimental and Clinical Traumatology, AUVA Research Center for Traumatology, 1200 Vienna, Austria; ^3^Department of Traumatology, Hand-, Plastic-, and Reconstructive Surgery, University Hospital Ulm, 89075 Ulm, Germany

## Abstract

Multiorgan failure (MOF) represents the leading cause of death in patients with sepsis and systemic inflammatory response syndrome (SIRS) following severe trauma. The underlying immune response is highly complex and involves activation of the complement system as a crucial entity of innate immunity. Uncontrolled activation of the complement system during sepsis and SIRS with in excessive generation of complement activation products contributes to an ensuing dysfunction of various organ systems. In the present review, mechanisms of the inflammatory response in the development of MOF in sepsis and SIRS with particular focus on the complement system are discussed.

## 1. Introduction

In the 1970s, a syndrome of progressive, sequentially dysfunctional organ systems has been firstly characterized, eventually referred to as multiorgan failure (MOF) [1, 2]. As a predominant underlying condition, sepsis and sepsis-associated MOF represent one of the leading causes of death of hospitalized patients with reported morality rates ranging from 28% to 56% [3, 4]. Likewise, severe trauma and trauma-related multiorgan failure remain the leading cause of death in people below the age of 40 [5, 6]. 

The conception of organ failure has changed over the years and various scoring systems for the classification and diagnosis of MOF exist all of which attempt to quantify the degree of organ failure [7–9]. Currently, MOF is regarded as a continuous process of varying levels of organ failure rather than an all-or-none event [10]. To characterize MOF, six different organ systems are regarded as “key organs”: lungs, cardiovascular system, kidneys, liver, coagulation system, and central nervous system.

Depending on the severity and various predisposing conditions, the initial insult (tissue trauma, infection) can induce a systemic host response that is characterized by the release of pro- and anti-inflammatory cytokines and metabolites (e.g., reactive oxygen (ROS) and nitrogen species (NOS)), activation of plasmatic cascade systems, such as the complement and the coagulation systems, and the appearance of acute phase proteins as well as hormonal and neuronal mediators [11–13]. Imbalanced systemic immune responses can ultimately lead to accumulation of leukocytes, disseminated intravascular coagulation (DIC), and microcirculatory dysfunction with subsequent apoptosis and necrosis of parenchymal cells, finally resulting in the development of MOF [12, 14, 15].

As a central entity of innate immunity, the complement system is immediately activated after trauma or infection in order to control the replication of intruding pathogens. In humans, the plasma levels of complement activation products rise early, are persistently elevated in patients after thermal injury, trauma, and sepsis, and correlate with the severity of injury and inversely with the outcome [16–22]. It is well established that activation of the complement cascade alters functional responses of neutrophils (PMN) in the course of systemic inflammation and contributes to the development of organ failure [15, 23]. In experimental sepsis, the blockade of complement anaphylatoxin C5a virtually prevented the appearance of MOF and improved the outcome [24–26]. Previous studies strongly suggest a mutual crosstalk between the complement and the coagulation system [27–30]. Due to the complex nature of plasmatic cascades and their interconnections, the role and regulations of the complement system, especially in states of disease, are still inadequately understood.

 This article is sought to provide insights into the pathogenesis of multiorgan failure associated with systemic inflammation with particular focus on the role of the complement system. Furthermore, potential therapeutic strategies targeting the complement cascade to prevent the development of MOF as well as possible future research directions are addressed.

## 2. Pathways of Complement Activation 

The complement system can be activated via four different pathways, the classical, the alternative, and the lectin pathway [31–33]. All three pathways lead to the assembly of the C3 convertase which cleaves C3 into C3a and C3b [31, 32]. Incorporation of C3b into the C3 convertase results in formation of the C5 convertase, which cleaves C5 into C5a and C5b. The split products C3a and C5a act as potent anaphylatoxins. C3b is an important opsonic factor, while C5b initiates the formation the membrane attack complex (C5b-9). In addition, various non-complement serine proteases seem to cleave complement components into biologically active complement products with variable efficacy [34]. In particular, thrombin has been found to function as a C5-convertase that does not require the presence of C3 or C3b [28]. Moreover, proteases from PMN and macrophages can cleave C5 as well [35, 36].

There is evidence that all three complement activation pathways are activated in SIRS and sepsis. Interestingly, it has been demonstrated that during the course of sepsis alternative pathway activation occurs earlier than activation of the classical pathway [37]. Based on their distinct mechanisms and kinetics of activation, it has been hypothesized that classical pathway activation in sepsis plays a crucial role in the clearance of pathogenic factors, while the alternative pathway is thought to be essential for fighting against infections by invading microorganisms [38]. Although the knowledge about the underlying mechanisms is limited, recent reports suggest a particular role of mannose-binding lectin (MBL) and the lectin pathway in the development of MOF. In sterile systemic inflammation (systemic inflammatory response syndrome, SIRS), patients with functional MBL deficiency due to MBL consumption did not develop MOF unless MBL was reconstituted by transfusion of fresh frozen plasma [39]. In contrast, septic patients with MBL depletion showed significantly higher sequential organ failure assessment (SOFA) scores, whereas functional MBL levels and activity in sepsis were associated with moderate SOFA scores and better prognosis [40], suggesting that MBL might be essential for defence against infections on the one hand, but might also harm the host and contribute to the development of MOF on the other hand. Therefore, as indicated by this dual function of the lectin pathway, the role of the complement system in systemic inflammation sometimes is referred to as a double-edged sword. 

## 3. Dysfunction of the Central Nervous System

**Figure 1:**
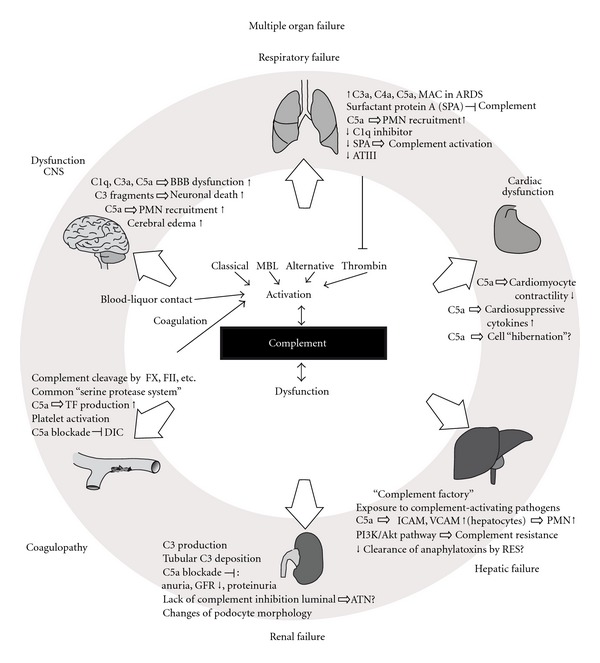
Summarizing illustration on the effects of excessive complement activation on various organ systems and the development of organ failure. For details see text. MBL: mannose-binding lectin, CNS: central nervous system, BBB: blood brain barrier, PMN: polymorphonuclear neutrophils, ARDS: acute respiratory distress syndrome, ATIII: antithrombin III, RES: reticuloendothelial system, GFR: glomerular filtration rate, ATN: acute tubular necrosis, FX: coagulation factor X, FII: coagulation factor II, TF: tissue factor, DIC: disseminated intravascular coagulation.

Historically, the central nervous system (CNS) was defined as an “immunological privileged organ” because of its separation from peripheral circulation by the blood-brain barrier (BBB). However, it became evident that the CNS is a rich source of inflammatory mediators and complement proteins can be produced by neurons, astrocytes, microglia, and oligodendroglia [41–43]. Severe trauma and sepsis are associated with systemic inflammation that can lead to blood-brain barrier (BBB) dysfunction and cerebral edema, regardless of the presence of traumatic brain injury (TBI) [44]. The breakdown of the BBB is considered to be a key event in the development of septic encephalopathy, while the cellular and molecular mechanisms of sepsis-induced brain damage are still vastly unknown [45]. Interestingly, the direct contact between blood and cerebrospinal fluid leads to complement activation, and the extent of intrathecal complement activation is associated with BBB dysfunction [46]. In addition, intracerebral complement levels increase under pathological conditions due to leakage of serum-derived complement proteins into the subarachnoidal space after breach of the BBB as well as increased complement biosynthesis in the CNS [47]. C1q, C3a, and C5a contribute to intracranial inflammation by induction of BBB damage and increase in vascular permeability [47, 48]. Blood-derived leukocytes, predominantly PMN, are then able to transmigrate into the CNS and release proteases and free radicals resulting in tissue damage (Figure 1) [47, 49]. In line with this, in experimental sepsis blockade of C5a attenuated pathophysiological changes that are typically associated with septic encephalopathy [50]. C3 and its derivates seem to play a central role in the pathogenesis of CNS dysfunction. Accumulation of C3 fragments is related to neuronal cell death and intracerebral PMN infiltration [51]. Previous studies suggested that the alternative pathway activation is a leading mechanism for neuronal cell death after closed head injury [52, 53]. C5a can induce neuronal apoptosis via the interaction with its receptor (C5aR), which is abundantly expressed on various cell types in the CNS [54, 55]. Finally, inactivation of the complement regulatory proteins on neurons during inflammation pave the road for complement-mediated lysis of homologous cells by the membrane attack complex [56]. Despite the unambiguous involvement in various pathological mechanisms, the role of the complement system in the pathogenesis of CNS dysfunction appears to be a double-edged sword since it has been reported that C3a as well as C5a also may mediate neuroprotective and neuroregenerative effects [57, 58]. 

## 4. Respiratory Failure

Respiratory failure or acute respiratory distress syndrome (ARDS) represents a frequent complication after burn injury, multisystem trauma, shock, and systemic inflammation [59–61]. Although the liver represents the main source for the production of complement proteins, virtually all complement proteins can be locally produced in the lung by type II alveolar pneumocytes, alveolar macrophages, and lung fibroblasts [62–64]. While the total pulmonary complement protein concentration is at comparable levels as found in serum, its activity in normal lung is markedly reduced which is attributed to the ability of surfactant protein A (SPA) to inhibit complement [65, 66]. In various studies, patients with ARDS showed evidence for robust complement activation, the extent of which correlated with the degree and outcome of ARDS [67, 68]. In particular, the complement anaphylatoxin C5a and the MAC are in the focus of ARDS pathophysiology, but also elevated levels of C3a and C4a have been linked to the development of ARDS [68–72]. C5a promotes inflammation by causing extensive influx of activated PMN into lung tissue and the alveolar space and by enhancement of the early cytokine response (reviewed in [72, 73]). However, only little is known about the local regulation of complement activation. Besides, the complement inhibitory function of SPA, C1 inhibitor, which inhibits classical pathway activation, has been detected in human bronchoalveolar lavage fluids [65, 66]. Lung activity of both, surfactant protein and C1 inhibitor, is significantly reduced in patients with trauma-related ARDS [74, 75]. Beside complement activation, ARDS is accompanied by tissue factor generation and widespread pulmonary fibrin deposition [76, 77]. Here, antithrombin III (ATIII), which inhibits activated proteases including thrombin, seems to play a central role since ATIII levels inversely correlate with the outcome in the setting of sepsis, and ATIII has been shown to block the thrombin pathway of complement activation in a murine model of acute lung injury (Figure 1) [28, 78, 79]. In conclusion, systemic inflammation provokes local imbalances of the complement and the coagulation cascade shifting the lung equilibrium to a proinflammatory and procoagulant state, which then stimulates accumulated leukocytes to locally release cytokines, enzymes, and radicals that promote the classical features of ARDS. 

## 5. Cardiac Dysfunction

Heart dysfunction during inflammatory states shows a biphasic process with an early hyperdynamic phase followed by a pivotal hypodynamic phase [80]. Hallmarks of the hypodynamic phase are decreased cardiac output, reduced microvascular flow, and increased peripheral vascular resistance with rising plasma levels of catecholamines. It has been suggested that these changes initiate the vicious circle of multiorgan failure due to compromised organ perfusion, decreased oxygen and nutrient supply, and ischemia [81]. Various myocardial depressant factors that collectively trigger cardiac contractility deficits in systemic inflammation have been described, but no single agent responsible for myocardial dysfunction could be identified [81–87]. In previous reports, complement activation has been linked to hemodynamic depression, but the mechanisms by which complement activation products might cause dysfunction of cardiomyocytes remain to be defined in detail [81, 88, 89]. In experimental studies, C5a has been demonstrated to induce cardiac dysfunction with impaired cardiomyocyte contractility, which could be restored by blockade of C5a [90, 91]. But it is far from certain if C5a-C5aR interaction directly causes cellular alterations in cardiomyocytes that lead to impaired calcium handling, oxygen and ATP depletion, and loss of mitochondria with energy deficit [92, 93]. Recent research suggests that C5a causes the local release of cardiosuppressive cytokines and chemokines in cardiomyocytes eventually leading to cardiac dysfunction [94]. But it is also conceivable that complement anaphylatoxins contribute to induce “hibernation” in cardiomyocytes as it occurs in the response of the myocardium to ischemia [95]. In the ischemic heart, it is a common observation that the induction of contractile dysfunction by C5a is not a direct effect but rather involves secondary production of mediators (e.g., arachidonic acid metabolites), which then act on target cells (Figure 1) [96]. Further, predominantly the classical and the alternative pathway are activated upon myocardial ischemia. Treatment with C1 inhibitor or soluble complement receptor 1 has cardioprotective effects by suppression of adhesion molecule expression (p-selectin, ICAM-1), blockade of C3 deposition and its activity on cardiomyocytes, and by anti-apoptotic activity [97–100]. However, it remains to be evaluated whether similar events de facto occur in cardiac dysfunction during systemic inflammation.

## 6. Hepatic Failure

 The liver represents the “major production facility” for most complement proteins found in the blood compartment except C1q, factor D, and properdin [101]. Because of its integral role in metabolism and host defense, the liver plays a key role in the initiation of MODS [102, 103]. Enhanced interaction of leukocytes with hepatic endothelial cells and hepatic microperfusion disorders are fundamental contributors to liver failure during sepsis [104]. Like in other organs, complement activation products are generated among other inflammatory mediators during systemic inflammation, which initiate a cascade of intracellular events in target cells leading to upregulation of adhesion molecules (ICAM-1, VCAM-1) on hepatic epithelial cells, increase of vascular permeability, and priming and influx of leukocytes [104, 105]. Treatment with C1 inhibitor reduced VCAM expression and hepatic leukocyte adhesion in experimental acute hepatic failure, even after delayed injection [104]. Besides this mechanism, PMN mediate parenchymal damage after accumulation in sinusoids, which does not depend on cellular adhesion molecules [106]. The liver is not only the main source of complement proteins but is also constantly exposed to complement-activating pathogens via the portal venous system [107, 108]. Immune complexes, anaphylatoxins, and activated complement components are cleared from circulation by the reticuloendothelial system lining the sinusoids without being detriment to hepatic function [101]. However, the efficiency of the reticuloendothelial system does not suffice to protect the liver. Therefore, hepatocytes are endowed with a unique mechanism to protect themselves from complement-induced cytotoxicity [107]. It is intriguing that this protection is not dependent on the complement regulatory proteins on the cell surface [107]. Instead, the inurement of hepatocytes to complement and its activated products requires the integrity of the PI3K/Akt pathway [107]. In turn, the PI3K/Akt pathway supposedly controls C5a-mediated effects in PMN and monocytes [109]. In experimental sepsis, anti-C5a treatment circumvented the development of MOF and attenuated markers of acute hepatic failure (e.g., bilirubin, ALT, AST, LDH) (Figure 1) [24]. Thus, it is tempting to speculate that under conditions, in which C5a is systemically generated, impairment of the PI3K/Akt pathway may lead to increased susceptibility for complement-mediated cytotoxicity of hepatocytes and subsequent organ failure. On the other hand, a potential role for C5a in tissue repair has been suggested [73].

## 7. Renal Failure

Acute renal failure (ARF) is hallmarked by abrupt decline in glomerular filtration and acute tubular necrosis in association with the appearance of multiple inflammatory mediators [110–112]. In sepsis, ARF occurs already at modest levels of hypotension suggesting that other mechanisms than ischemia are involved [110]. Like in parenchymal cells of lung and brain, complement proteins can be locally produced by renal cells, such as proximal tubular cells, *in vitro* and *in vivo* [113, 114]. In the case of C3, there is evidence that its renal production even contributes to the circulating C3 pool [115]. Proximal tubular cells are capable of activating the alternative pathway, terminating in the binding of MAC to the cell surface [116]. In this context, it is of particular interest that the luminal brush border lacks complement regulatory proteins on the cell surface [117]. Under certain circumstances, paucity of protection against complement-mediated cell lysis predisposes to tubular damage due to the luminal deposition of filtered complement components [118]. The deposition of C3 and C4 is well established in glomerular disease, but only C3 deposition, and no evidence for C4 deposition, along tubules could be found in acute tubular necrosis after renal ischemia/reperfusion injury, indicating that the alternative pathway is the predominant complement activation pathway for the development of acute tubular necrosis [118]. However, suppression of C3 activation failed to affect the degree of ARF in a murine model of systemic inflammation, although C3 synthesis was upregulated, resulting in basolateral tubular C3 deposition [110]. In disagreement with these authors' conclusion, this does not necessarily mean that complement is not responsible for ARF in the setting of systemic inflammation since it is now known that the downstream complement cascade can be activated despite the absence of C3 [28]. In contrast, the occurrence of ARF could be clearly linked to the generation of C5a during experimental sepsis, and parameters of ARF (creatinine, urine output, glomerular filtration rate, proteinuria) as well as morphological changes of podocytes were greatly attenuated by anti-C5a treatment [24]. Beyond their local inflammatory and chemotactic features, C3a and C5a have vascular effects that contribute to changes in renal hemodynamics in ARF (Figure 1) [119]. Taken together, the complement system represents a key effector of ARF by a variety of mechanisms, which affect renal perfusion and glomerular filtration as well as tubular function.

## 8. Dysregulation of the Coagulation System

The coagulation system and the complement system are both proteolytic cascades composed of serine proteases that share structural characteristics. As descendants of a common ancestor, both systems can be basically activated by similar stimuli [120, 121]. Trauma and tissue injury often cause damage of the vasculature and subsequent bleeding, which is also associated with the risk of infection by intruding microorganisms [11]. Activation of both cascades is intended to occur locally under thorough regulations, but under certain circumstances, loss of control can lead to systemic activation with harmful consequences for the host [29]. Disseminated intravascular coagulation (DIC) represents a frequent complication after trauma, systemic inflammation, and sepsis [122, 123]. After the initial phase of hypercoagulability with intra- and extravascular fibrin clots, consumption of coagulation factors and dysfunction of thrombocytes can lead to hemorrhagic diasthesis and diffuse bleeding [79, 122, 123]. Intravascular fibrin clots are finally responsible for impaired microcirculation and hypoxic cellular damage [79]. Trauma, thermal injury, and infection predispose to thrombosis and the development of DIC and trigger the inflammatory response including complement activation, which, in turn, can trigger coagulation and vice versa [121, 123]. As mentioned above, thrombin is capable of cleaving C5, resulting in the generation of C5a. This concept of a direct crosstalk between central components of the complement and coagulation cascades is corroborated by the findings of elevated thrombin-antithrombin (TAT) complexes in the clinical and experimental setting of multiple injury [34]. Beside the C5-convertase activity of thrombin, various factors of the coagulation and fibrinolysis system, including FXa, FXIIa, plasmin, and kallikrein, can cleave complement components or their fragments [28, 124–126]. On the other hand, the inflammatory response and the complement system in particular amplify coagulation by modification of phospholipid membranes required for the initiation of the tissue factor (TF) pathway, activation of platelets, and upregulation of TF expression [121]. Specifically, activation of C5 can increase TF expression on leukocytes and blockade of C5a-ameliorated DIC in a rodent model of sepsis [27, 127]. The procoagulant activities of complement are aggravated by inhibition of anticoagulant mechanisms, such as complex formation of C4b-binding protein with protein S  (PS), which results in a loss of PS cofactor activity for activated protein C (APC) [128]. In turn, the protein C anticoagulant pathway does not only function as a regulator of the coagulation cascade by degradation of FVa and FVIIIa, but also dampens the inflammatory response [121, 129]. Traditionally, complement and coagulation were described as separate cascades, only linked by the ability of FXIIa to activate the classical complement pathway [124]. However, it becomes now more and more evident that the convergence between both systems extends beyond the biochemical nature of serine proteases, and multiple mutual interconnections form a highly complex network (Figure 1) [29, 30, 34]. Understanding the interplay is important to breach the vicious circle of systemic inflammation in order to be able prevent life-threatening complications. 

## 9. Conclusions

 Based upon the current understanding, the general role of complement in the pathogenesis of MOF can be conceptualized as follows: After trauma, burn, or severe tissue injury, systemic intravascular activation of the complement system with apparent loss over the control mechanisms occurs. Complement activation products trigger a cascade of cellular events in endothelial cells resulting in upregulation of adhesion molecules, release of proinflammatory mediators, and increased vascular permeability. Leukocytes are attracted by complement anaphylatoxins to transmigrate into parenchyma of various organs after adhesion to endothelial cells and extravasation. Activated leukocytes release inflammatory mediators, enzymes, and free radicals that harm parenchymal cells. Local production and activation of complement proteins in combination with loss of protection against complement-mediated lysis aggravate the degree of tissue injury. Interaction with the coagulation cascade causes disseminated intravascular coagulation and compromised microcirculation, which then augments organ dysfunction by ischemia. All events of this vicious circle finally merge into apoptosis and necrosis of parenchymal cells with the development of multiple organ dysfunction syndrome. The complement anaphylatoxins C5a and C3a not only trigger the inflammatory response but also directly alter cellular functions of parenchymal cells as well as leukocytes by interaction with their specific receptors, which are abundantly expressed on numerous cell types. However, the organ-specific mechanisms and intracellular events that follow receptor binding, such as mitogen-activated protein kinase (MAPK) pathways, remain to be evaluated in future studies. As outlined above in the description of cardiac dysfunction, organ failure might reflect a cellular resting state, also described as hibernation, as a response to a proinflammatory environment with uncoupling of the respiratory chain and mitochondrial dysfunction. However, it is not clear yet if and to which extent complement activation contributes to the pathophysiology of hibernation in human cells. 

Since complement activation occurs as a rapid event after the initial insult, it appears auspicious to use intervention in the complement system as a therapeutic approach in order to prevent the development of MOF. Strategies to inhibit complement include (i) the application of endogenous complement inhibitors (C1 inhibitor, soluble complement receptor-1) [130], (ii) administration of antibodies or antagonists which block key proteins (C3, C5) of the complement cascade or neutralize complement-derived anaphylatoxins (C3a, C5a) [25, 131], and (iii) interference of C5a, C3a interaction with their receptors by receptor-specific antagonists [26]. In addition, upregulation or incorporation of membrane-bound complement-regulatory proteins could protect organs from complement-mediated cytotoxicity. Protection against complement-mediated inflammatory tissue damage could be achieved in various experimental settings. However, total blockade of the complement cascade might impair the capability to clear invaded pathogens and increase the risk of infection. Therefore, targeting the complement system in inflammation should rather aim to balance or control its activation with suppression of the harmful effects, but without detriment of the protective and reparative complement functions.
